# A homozygous missense mutation in the fibroblast growth factor 5 gene is associated with the long-hair trait in Angora rabbits

**DOI:** 10.1186/s12864-023-09405-2

**Published:** 2023-06-02

**Authors:** Nazira Fatima, Linying Jia, Baoning Liu, Lu Li, Liang Bai, Weirong Wang, Sihai Zhao, Rong Wang, Enqi Liu

**Affiliations:** 1 Department of Laboratory Animal Science, School of Basic Medical Sciences, Xi’an Jiaotong University, Xi’an, 710061 Shaanxi China; 2 Laboratory Animal Center, Xi’an Jiaotong University, Xi’an, 710061 Shaanxi China

**Keywords:** Selective sweeping, Single-nucleotide polymorphism (SNP), Fibroblast growth factor 5 gene (*Fgf5*), Long hair trait, Rabbit

## Abstract

**Background:**

Rabbits are well-domesticated animals. As a crucial economic animal, rabbit has been successfully bred into wool-use, meat-use and fur-use breeds. Hair length is one of the most economically important traits affecting profitability in wool rabbits. In this study, to identify selection signatures with the long-hair trait, whole-genomic resequencing of long-haired rabbits (Angora rabbits) and short-haired rabbits (Rex and New Zealand rabbits) was performed.

**Results:**

By genome-wide selective sweeping analysis based on population comparison, we identified a total of 5.85 Mb regions (containing 174 candidate genes) with strong selection signals. Six of these genes (*Dusp1*, *Ihh*, *Fam134a*, *Map3k1*, *Spata16*, and *Fgf5*) were enriched in the MAPK signalling and Hedgehog signalling pathways, both of which are closely associated with hair growth regulation. Among these genes, *Fgf5* encodes the FGF5 protein, which is a well-established regulator of hair growth. There was a nonsynonymous nucleotide substitution (T19234C) in the *Fgf5* gene. At this locus, the C allele was present in all of the tested Angora rabbits, while the T allele was dominant in New Zealand and Rex rabbits. We further confirmed that the C allele was conserved in Angora rabbits by screening an additional 135 rabbits. Moreover, the results of functional predictions and co-immunoprecipitation revealed that the T19234C mutation impaired the binding capacity of FGF5 to its receptor FGFR1.

**Conclusions:**

We discovered that the homozygous missense mutation T19234C within Fgf5 might contribute to the long-hair trait of Angora rabbits by reducing its receptor binding capacity. This finding will provide new insights into the genetic basis underlying the genetic improvement of Angora rabbits and benefit the improvement of rabbit breeding in the future.

**Supplementary Information:**

The online version contains supplementary material available at 10.1186/s12864-023-09405-2.

## Background

Rabbit domestication was initiated in monasteries in southern France as recently as ~ 1500 years ago [1]. According to exceptionally high phenotypic diversity, more than 300 breeds have been recognized worldwide [2]. As one of crucial economic animals, domestic rabbit populations have been mainly bred for three purposes: wool production, meat production and fur production.

Angora rabbit is a famous breed of wool rabbit and is largely bred for its high yield and quality of wool. Approximately 10,000 tons of Angora wool are produced every year, which makes it the third most-produced animal fibre in the world, after sheep wool and mohair [3, 4]. The wool of Angora rabbits is characterized by long, slender, smooth, soft, fluffy and warm fibres. Therefore, the wool of Angora rabbit is commonly used in apparel such as sweaters and suitings, knitting yarn, and felting. For Angora rabbits, the active phase of hair growth is significantly longer than that of short-haired rabbits, such as New Zealand rabbits and Rex rabbits [2]. The mechanisms regulating hair traits are complicated. Many molecules and signalling pathways are involved in regulating the hair growth cycle, such as fibroblast growth factor (FGF), transforming growth factor (TGF)-β, Sonic Hedgehog (Shh) signalling and MAPK signalling [5–7].

Phenotype is closely correlated with genotype. The variation in genotype is able to result in notable changes in phenotype, such as in behaviour, morphology, physiology, and reproduction. To further identify the genetic changes underlying the long-hair phenotypic developments of Angara rabbits, we used the whole genome sequencing approach to explore the genetic differences between long-haired rabbits (Angora rabbit) and short-haired rabbits (New Zealand rabbit and Rex rabbit) and identified genetic components related to the long-hair phenotypic trait. The results will guide us to improve rabbit breeding in the future.

## Results

### Sequencing, mapping, SNP calling

**Fig. 1:**
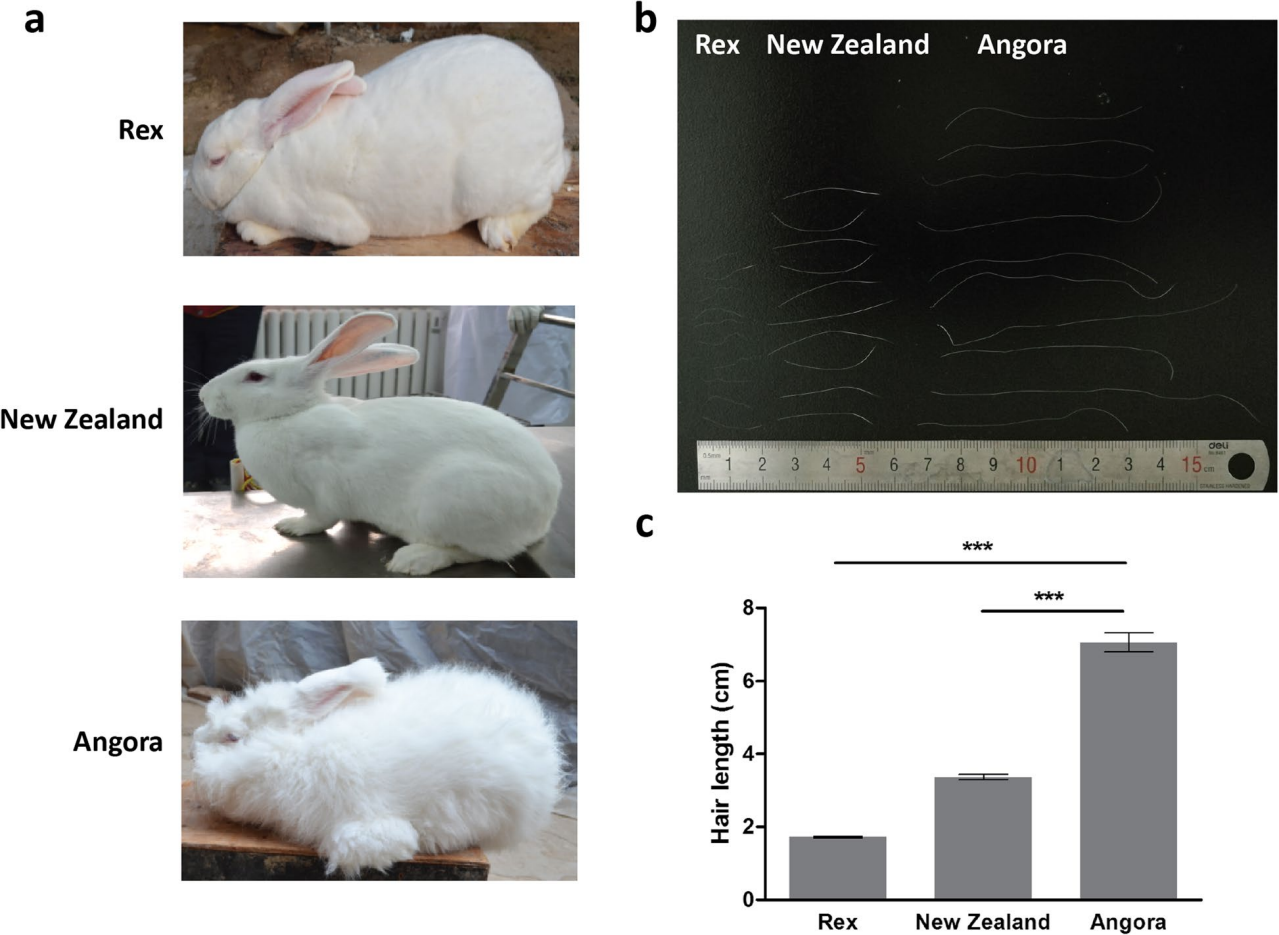
Long-hair phenotype in Angora rabbits. **a** The appearance of three breeds of rabbit. **b** Variations in hair length among Rex, New Zealand and Angora rabbits. **c** Statistics on hair length of the three rabbit breeds. The significance of the difference between Angora and Rex, as well as Angora and New Zealand, is calculated by Student’s *t* test, ** represents *p* < 0.01

Three populations of domestic rabbits (Angora, New Zealand and Rex) were involved in this study (Fig. 1a). Each population included 6 female rabbits selected from different farms in China. There was great variance in the hair length among the three breeds, and the average values of the hair length were approximately 1.7, 3.4 and 7.0 cm for Rex, New Zealand and Angora, respectively (Fig. 1b, c).

Sequencing of 18 individuals generated 320.09 Gb of paired-end DNA sequence, of which 314.62 Gb (~ 98.29%) of effective reads were mapped to the rabbit reference genome assembly (Orycun 2.0) (Table 1). For each individual, ~ 98.38% of reads mapped to 97.89% of the reference genome assembly with a 6.57-fold average depth (Table 1).

**Table 1:** Summary and mapping statistics of selected domestic rabbits

Sample name	Raw Base(bp)	Effective Rate(%)	Mapped reads	Total reads	Mapping rate(%)	Average depth(X)	Coverage 1X(%)
Angora_1	17,119,857,250	97.61	131,343,732	133,723,114	98.22	6.29	97.69
Angora_2	17,425,961,500	98.29	134,957,371	137,010,030	98.5	6.46	97.69
Angora_3	17,574,152,750	98.2	135,443,645	137,771,608	98.31	6.48	97.65
Angora_4	17,258,029,750	97.94	132,950,495	135,216,400	98.32	6.36	97.68
Angora_5	17,370,164,250	98.62	134,813,390	136,932,174	98.45	6.43	98.25
Angora_6	19,624,237,750	98.79	152,844,924	155,093,428	98.55	7.28	98.23
New Zealand_1	17,276,228,250	98.04	133,413,523	135,650,682	98.35	6.39	97.64
New Zealand_2	18,211,094,500	98.37	140,666,365	143,137,310	98.27	6.72	97.94
New Zealand_3	17,840,751,000	98.66	138,626,184	140,714,772	98.52	6.61	98.02
New Zealand_4	17,206,992,000	98.58	133,417,312	135,597,100	98.39	6.37	97.94
New Zealand_5	16,737,332,250	98.56	129,490,187	131,975,650	98.12	6.22	97.51
New Zealand_6	19,740,318,000	98.81	153,701,118	156,047,852	98.5	7.32	98.17
Rex_1	17,600,293,000	98.17	135,831,383	138,045,552	98.4	6.51	97.54
Rex_2	17,661,203,000	97.8	135,686,374	138,018,346	98.31	6.5	97.75
Rex_3	16,627,732,000	97.85	128,302,425	130,183,248	98.56	6.15	97.44
Rex_4	17,415,676,000	97.79	133,653,166	136,104,786	98.2	6.39	97.85
Rex_5	18,661,197,250	98.52	144,774,198	147,082,754	98.43	6.88	98.47
Rex_6	18,735,044,000	98.65	145,584,115	147,848,336	98.47	6.91	98.57
**total**	320,086,264,500	-					
**Average**	17,782,570,250	98.29	137,527,773	139,786,286	98.38	6.57	97.89

We performed single-nucleotide polymorphism (SNP) calling and identified 24.83 million (M) SNPs from these 18 individuals. Of these, 161,407 coding SNPs leading to 57,721 nonsynonymous nucleotide substitutions (57,050 missense, 607 stop gain and 64 stop loss) were detected (Table 2a). According to breeds, we pooled the SNPs into three groups, including 15.62 M from the 6 Angara rabbits, 16.95 M from the 6 New Zealand rabbits, and 16.54 M from the 6 Rex rabbits. 12.23 M of 24.83 M (49.25%) SNPs were shared among the three groups (Table 2b).

**Table 2:** Summary and annotation of SNPs on a population-scale

**a**				**b**				
**Category**		** Number of SNPs**		**Category**	**Angara**	**New Zealand**	**Rex**	**Total**
**Total**			24,831,754	Sample size	*n* = 6	*n* = 6	*n* = 6	*n* = 18
**Upstream**			156,603	Number of total SNPs	15,622,108	16,948,460	16,539,048	24,831,754
**Exonic**	Stop gain		607	Number of shared SNPs	12,234,559			
	Stop loss		64					
	Synonymous		103,686					
	Nonsynonymous		57,721	‘Upstream’ refers to a variant that overlaps with the 1 kb region upstream of the gene start site ‘Stop gain’ means that a nsSNP leads to the creation of a stop codon at the variant site ‘Stop loss’ means that a nsSNP leads to the elimination of a stop codon at the variant site ‘Splicing’ means that a variant is within 2 bp of a splice junction ‘Downstream’ means that a variant overlaps with the 1 kb region downstream of the gene end site ‘Upstream/Downstream’ means that a variant is located in downstream and upstream regions (possibly for two different genes)				
**Intronic**			6,751,084					
**Splicing**			670					
**Downstream**			148,646					
**Upstream/downstream**			1729					
**Intergenic**			17,610,944					

### Genome-wide selective sweep signals

**Fig. 2:**
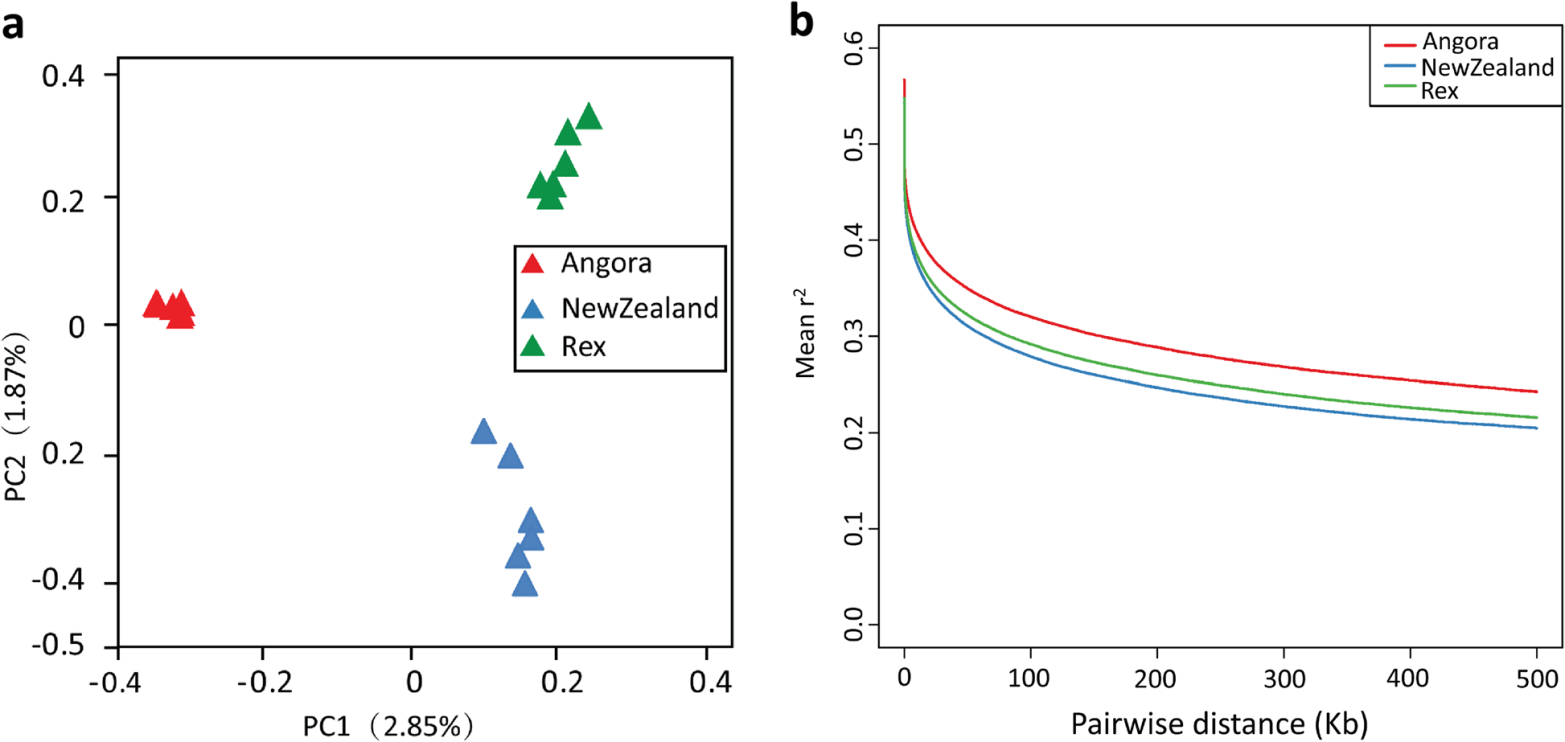
Analysis of population variation of the three rabbit breeds. **a** Principal component analyses (PCA) and (**b**) linkage-disequilibrium (LD) patterns of Angora, New Zealand and Rex rabbits

To clearly reveal the linkage between genotype and phenotype during rabbit artificial selection, we measured the genome-wide variations among Angora, New Zealand and Rex rabbits. Principal component analysis (PCA) revealed genetic separation among the three rabbit breeds (Fig. 2a). Moreover, Angora rabbits have the lowest levels of linkage disequilibrium (LD) across the range of distances separating loci, followed by Rex. New Zealand rabbits showed a relatively higher LD than the other two breeds (Fig. 2b). The results indicated that Angora rabbits suffered stronger selection pressure under artificial breeding programs, which led to lower genomic diversity in the Angora population.

**Fig. 3:**
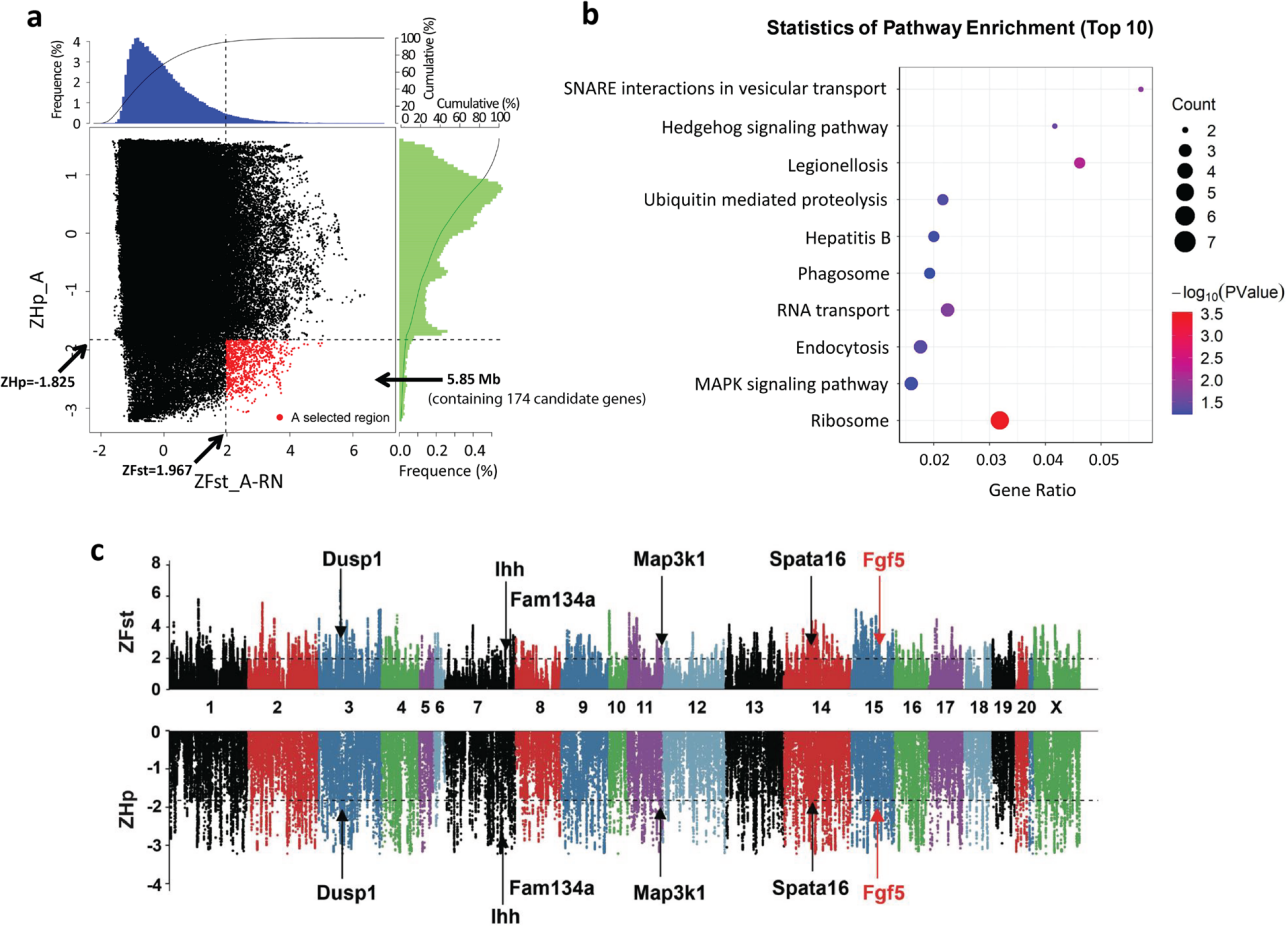
Selective sweep analysis of the rabbit genome. **a** Identification of genomic regions with strong selective sweep signals in Angora rabbits. Distribution of Angora ZHp values (ZHp_A) and ZFst values of Angora *vs.* New Zealand and Rex (ZFst_A-RN), which were calculated in 100 kb windows sliding in 10 kb steps. Data points located to the right of the vertical dotted lines (corresponding to 5% right tails of the empirical ZFst distribution) and below the horizontal dotted line (5% right tail of the empirical ZHp distribution) are identified as selected regions (red points). **b** KEGG enrichment analysis for the identified candidate genes. The top 10 enrichment pathways are presented. **c** Manhattan plot of selective sweeps in Angora rabbits. The chromosome numbers are presented along the x-axes. Bins of ZFst and ZHp are presented along the y-axes. The Manhattan plots are pointed the value for each window of ZFst and ZHp. The horizontal dashed lines indicate the threshold at ZFst = 1.967 and ZHp = -1.825. Genes residing within the selected regions and associated with hair growth are indicated by their gene names

To detect signals of strong selection, we searched the Angora rabbit genome for regions with reduced heterozygosity (Hp_A) and increased genetic distance to the short-haired rabbits (New Zealand rabbit and Rex rabbit) (Fst_A-RN). We calculated the Hp_A value and Fst_A-RN value, which were Z-transformed to ZHp_A and ZFst_A-RN, respectively. Then, we determined the selective sweep regions by using an empirical procedure and selected windows simultaneously with significantly low ZHp values (5% right tail, where ZHp_A = -1.825) and significantly high ZFst values (5% right tail, where ZFst_A-RN = 1.967) of the empirical distribution as regions with strong selective sweep signals along the genome (Fig. 3a), which should harbour genes that underwent selective sweep. Consequently, we identified a total of 5.85 Mb regions (containing 174 genes) with strong selective sweep signals in Angora rabbits (Fig. 3a). The 174 genes were considered as the candidate genes, which were enriched in a total of 93 pathways during KEGG pathway enrichment analysis (Table S1). In the top 10 enrichment pathways, some were closely associated with hair growth regulation, such as the MAPK signalling pathway and Hedgehog signalling pathway (Fig. 3b). Six candidate genes (*Dusp1*, *Ihh*, *Fam134a*, *Map3k1*, *Spata16* and *Fgf5*) were included in the two pathways (Fig. 3c). Among the six genes, we decided to further evaluate *Fgf5* because of the well-established biological significance of FGF5-mediated signalling for hair growth cycling regulation.

### The T19234C mutation in *Fgf5* is conserved in Angora rabbits

**Fig. 4:**
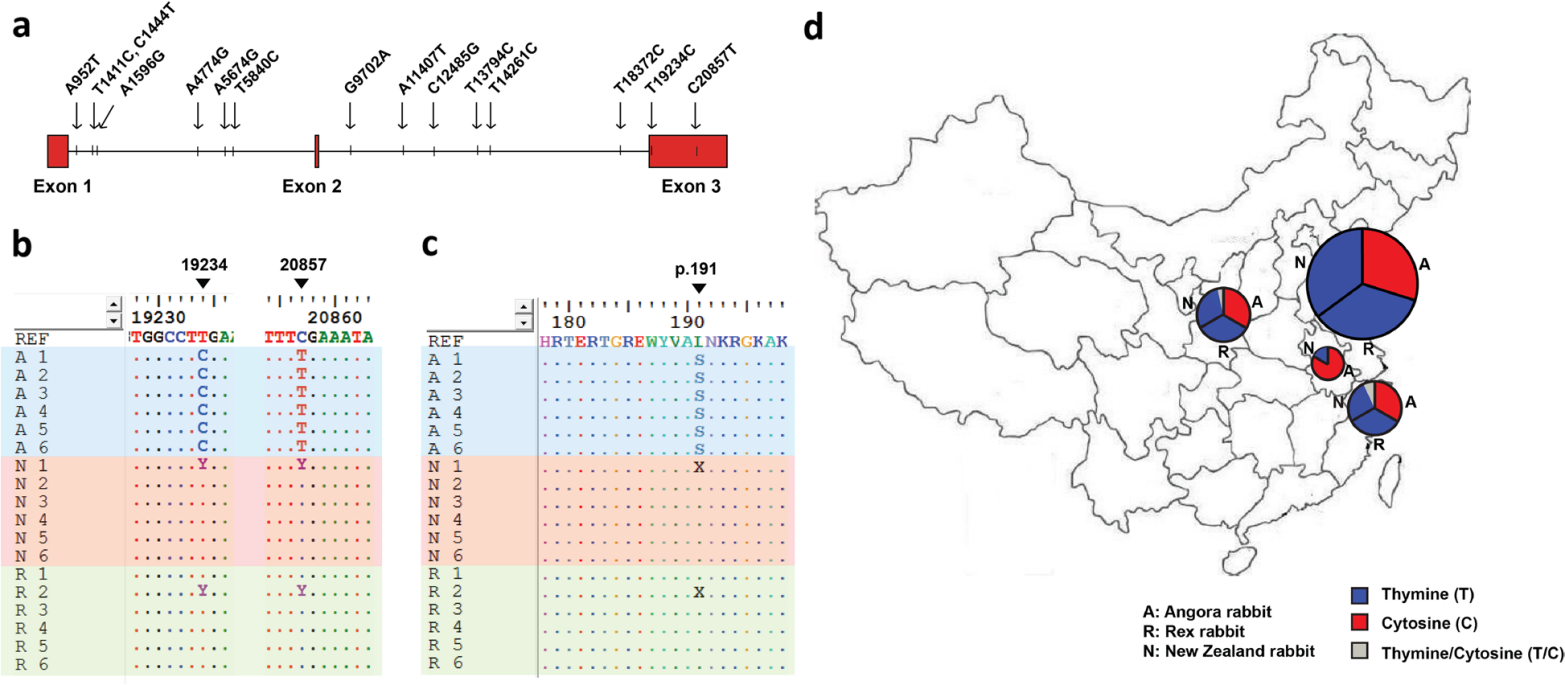
SNP analysis of the *Fgf5* gene in rabbits. **a** SNPs in *Fgf5*. A total of 15 SNPs were detected in *Fgf5*, the loci of which are indicated in the schematic structure of the *Fgf5* gene. **b** Alignment of the *Fgf5* nucleotide sequences around the 19,234 and 20,857 loci. *Fgf5* sequences of the eighteen rabbits were obtained from whole genome sequencing. The sequences of Angora (A1-A6), New Zealand (N1-N6) and Rex (R1-R6) are labelled with blue, red and green backgrounds, respectively. Dots indicate positions identical to the reference genome sequence (REF). Nucleotides inconsistent with the reference sequence are listed. The 19,234 and 20,857 loci of the *Fgf5* gene are indicated using black triangles. T: thymine, C: cytosine, Y: thymine/cytosine. **c** Alignment of the FGF5 amino acid sequences around residue 191. *Fgf5* coding sequences of the eighteen sequenced rabbits were translated into amino acid sequences, followed by sequence alignment. Dots indicate positions identical to the reference sequence (UniProtKB-G1T394). The missense mutations are shown. L: leucine, S: serine, X: leucine/serine. **d** Validation of the T19234C SNP by Sanger sequencing. EDTA-blood samples of 135 rabbits were collected from Shaanxi, Shandong, Jiangsu and Anhui provinces of China. The *Fgf5* partial fragment was amplified, followed by sequencing and alignment analysis. For each province, nucleotide statistics of the 19,234 locus are presented in a pie chart

The *Fgf5* gene resides on Chromosome 15 of rabbit and contains three exons. In this study, the *Fgf5* gene was included in the selective sweep regions. By further analyzing SNPs in the *Fgf5* gene, we found that there were a total of 15 SNPs; 13 SNP loci were located in introns, and 2 of them were present in extron 3 (Fig. 4a). Although 2 SNP loci existed in the extron, only T19234C resulted in a homozygous missense mutation (Fig. 4b), which converted the leucine at residue 191 to serine (p. L191S) (Fig. 4c). At the SNP locus (T19234C), the C allele was observed in all the tested Angora rabbits, while the T allele was dominant in the New Zealand and Rex rabbits, with a frequency of 83.3% (Fig. 4b).

To confirm that the T19234C mutation exists conservatively in Angora rabbits, Sanger sequencing was used to validate this mutation by screening an additional 135 rabbits (including 52 Angora, 43 New Zealand and 40 Rex rabbits) distributed in four major rabbit breeding provinces. The results showed that in Angora rabbits, the frequency of the C allele at this locus was 100%, but it was rare in New Zealand and Rex rabbits. Instead, the T allele was dominant in New Zealand and Rex rabbits, with frequencies of 93% and 100%, respectively (Fig. 4d). This result suggested that the T19234C mutation in the *Fgf5* gene might be correlated with the long-hair trait.

### The p. L191S weakened the interaction between FGF5 and its receptor FGFR1

**Fig. 5:**
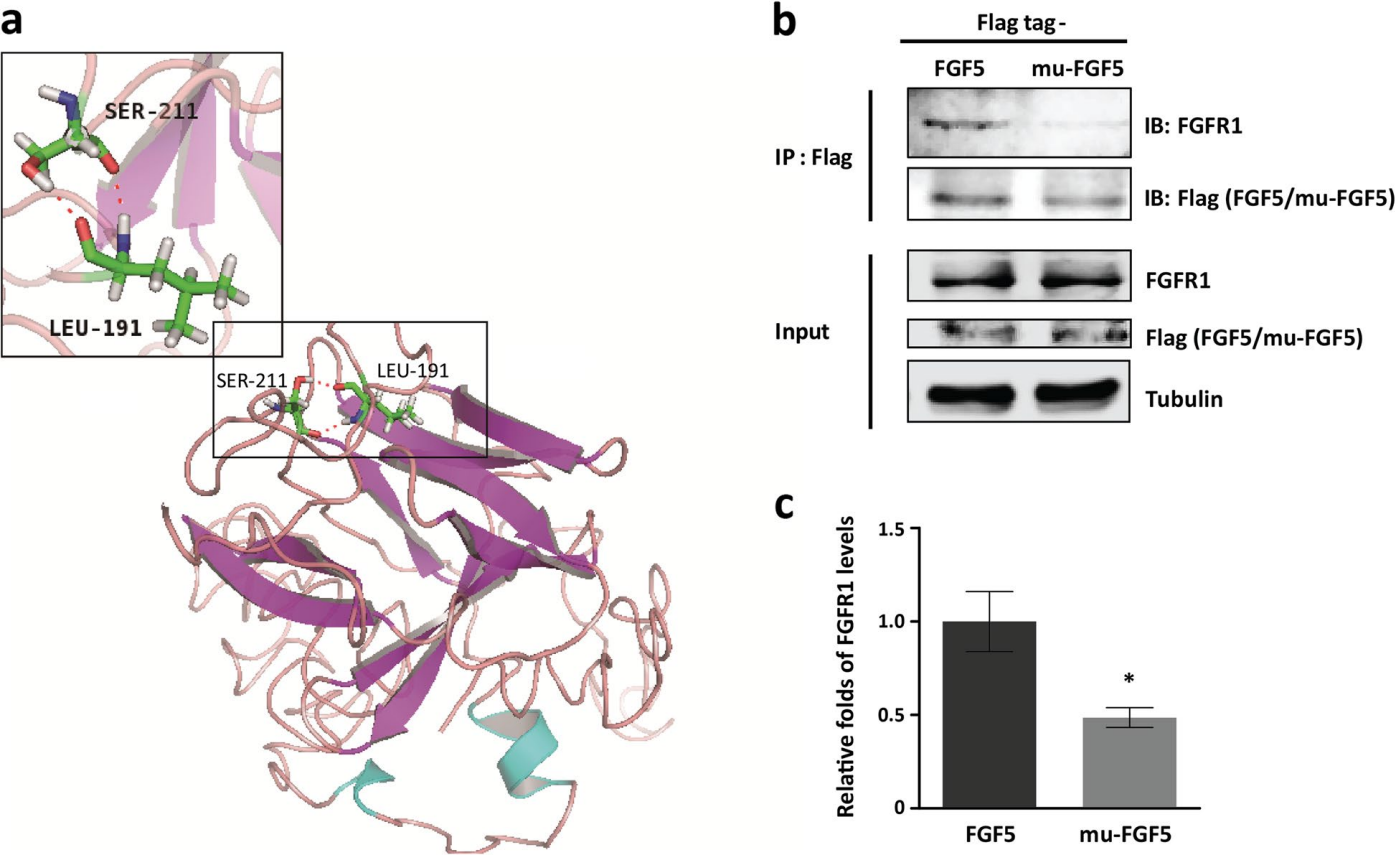
p. L191S mutation reduces the binding ability of FGF5 to FGFR1. **a** Structure prediction of FGF5. The three-dimensional structure of the FGF5 protein was predicted by I-TASSER based on its homologue, FGF1 (PDB ID: 3OJV). The predicted structure of FGF5 was visualized by PyMOL and shown as a ribbon diagram. The interactions between the side chains of L191 and S211 are magnified. **b** Evaluation of the effect of p. L191S mutation on the interaction between FGF5 and FGFR1. HEK293T cells were co-transfected with FGFR1 and FGF5-Flag or mu-FGF5-Flag plasmids. At 48 h after transfection, the cells were lysed with IP lysis buffer, followed by IP with anti-Flag IP resin. The input and IP products were subjected to immunoblotting with antibodies against FGFR1, Flag and tubulin. **c** Densitometry analysis of the digital immunoblotting images including Panel **b** and others of repeated tests. Relative fold changes in FGFR1 levels in IP products are shown after normalization to the corresponding FGF5 or mu-FGF5 level. Error bars represent standard errors of the repeated experiments. Significant differences between the two groups were calculated by Student’s *t* test, * represents *p* < 0.05

The hair growth cycle is typically divided into three phases: anagen (growing phase), catagen (regression phase) and telogen (resting phase) [7]. FGF5 is a crucial regulator of hair growth [8]. It has catagen-promoting activity to induce dermal papillae to finish anagen and to begin catagen [9]. FGF5 contains a conserved receptor binding domain whose coding sequence spans all three exons and interacts with its receptor, FGFR1 [9, 10]. FGF5 exerts its functions by binding with high affinity to FGFR1, which is expressed in the dermal papillae and regulates the development and activity of hair follicles [9, 10]. Due to the T19234C mutation leading to a missense mutation at residue 191 (p. L191S), we used the functional prediction programs PROVEAN [11] and Polyphen-2 [12] to determine the effect of the missense mutation. PROVEAN predicted the p. L191S mutation would be “deleterious” with a score of “-5.392”. Polyphen-2 predicted that the mutation would be “probably damaging” with a score of “1”. These results suggested that p. L191S was likely to have a negative impact on the function of FGF5. Moreover, we predicted and analysed the structure and ligand binding sites of FGF5 by the I-TASSER program [13]. The results showed that in FGF5, the L191 residue was adjacent to the ligand binding sites (94, 192, 193, 198, 209, 210, 211), and the L191 residue interacted with the S211 residue (Fig. 5a). Therefore, we speculated that substitution of the hydrophobic leucine with a hydrophilic serine at residue 191 was likely to impact the binding of FGF5 with its receptor FGFR1.

To determine the effect of p. L191S on the interaction between FGF5 and FGFR1, a point mutant of the FGF5 plasmid (mu-FGF5) was constructed. In mu-FGF5, nucleotide 572 within the coding sequence of *Fgf5* was mutated from T to C, which resulted in leucine at residue 191 being replaced by serine. The FGFR1 and FGF5 or mu-FGF5 plasmids were co-transfected into HEK293T cells. Then, co-IP was conducted to detect the binding ability of FGF5 and mu-FGF5 on FGFR1. The results showed that the binding of mu-FGF5 and FGFR1 was much weaker than that of FGF5 and FGFR1 (Fig. 5b). In comparison with FGF5, mu-FGF5 had a 0.49-fold decline in the binding ability to FGFR1 (Fig. 5c). This result indicated that the T19234C-induced p. L191S mutation could impair the interaction between FGF5 and FGFR1.

## Discussion

Hair fibre length is a critical economic trait for wool rabbits. Angora rabbit is a famous breed of wool rabbit for its high yield of long hair. In this study, based on next-generation sequencing data, we compared the genomes of Angora rabbit to the genomes of two breeds of short-haired rabbit: New Zealand and Rex rabbit. The SNPs and selective sweeping signals were exploited. Several genes associated with hair growth regulation, such as *Dusp1*, *Ihh*, *Fam134a*, *Map3k1*, *Spata16* and *Fgf5*, underwent strong selection sweeps. Moreover, we discovered that the T19234C mutation in *Fgf5* was conserved in Angora rabbits, which significantly impaired the binding capacity of FGF5 to its receptor FGFR1. This finding is beneficial for understanding the mechanism of hair development, thus promoting rabbit breeding.

Angora rabbit, the famous breed of long-haired rabbit, is widely bred all over the world. Its active phase of hair growth is double that of normal rabbits. The annual world production of angora wool is estimated at 2500 to 3000 tonnes a year, with approximately 90% of the supply produced in China [4]. In China, angora farms count more than 50 million rabbits, which are mainly indigenous Chinese breeds, including Zhexi Angora rabbit, bred from German angora. Zhexi Angora rabbits show an excellent capacity for wool production. The wool yield of one adult is approximately 2 kg per year. Currently, China exports approximately half of its production to Europe, Japan and the Republic of Korea [4]. In this study, Zhexi Angora rabbit was used as the representative of long-haired rabbit to compare the genomic character with the other two commonly used economic rabbit breeds: New Zealand and Rex. New Zealand rabbit is one of the meat-use rabbit breeds because of its high growth rate, high feed conversion and slaughter rate [14]. Rex rabbits, characterized by short dense plush velvet-like fur, are commonly used for fur production [15]. Both New Zealand and Rex show a short-hair appearance. Among the three breeds, the hair length is Angora > New Zealand > Rex. In addition, we found that Angora rabbits had the lowest levels of LD among the three breeds, which indicated that Angora rabbits suffered stronger selection pressure than the other two breeds under artificial breeding programs.

The hair growth cycle consists of three phases: anagen, catagen, and telogen [7]. In the anagen phase, hair follicles grow rapidly and subsequently synthesize hair. After hair reaches the longest length, the cycle transitions into the catagen phase, during which the active growth of a hair end and the hair converts to a club hair. Catagen is followed by a resting phase known as telogen. During this final stage of a hair’s lifecycle, the hair ceases to grow any further and becomes fully keratinized. Several signalling pathways are involved in the regulation of hair growth cycling, including the MAPK signalling pathway and Hedgehog signalling pathway [5, 7]. It has shown that the MAPK pathway is essential in the hair cycle and hair follicle stem cell quiescence [16], and its activation promotes the proliferation and differentiation of hair follicle stem cells [17] and increases the production of growth factors [18]. Sonic hedgehog (Shh) and its mediated signalling also play central roles in the development of hair follicles in the embryo, as well as in the regulation of the hair growth cycle in adults [19–22]. *Shh* mutant skin gives rise to large abnormal follicles, and no hair is formed [22]. Exogenously administered SHH in postnatal mice induces resting hair follicles to enter anagen and stimulates hair growth [19]. Conversely, treatment of adult mice with an antibody against SHH blocks the active phase of the hair growth cycle (anagen) and blocks hair regrowth [23]. In the present study, to explore the genetic basis for regulating the long hair trait of Angora, we conducted whole genome resequencing on Angora, New Zealand and Rex rabbits, followed by analysis of the strong selective sweep regions. We found that many genes with strong selective signals could be enriched in the MAPK and Hedgehog signalling pathways, such as *Dusp1*, *Ihh*, *Fam134a*, *Map3k1*, *Spata16*, and *Fgf5*. These results indicate that these genes may be involved in the development of the long-hair trait in Angora rabbits by regulating MAPK signalling and Hedgehog signalling.

In previous studies, RNA sequencing has been used to investigate the genetic principles of hair traits in rabbits. Messenger RNAs, long noncoding RNAs and microRNAs (miRNAs) have been discovered to control hair growth cycling by regulating protein-coding gene expression at the posttranscriptional and transcriptional levels [24–27]. In the present study, we used whole genome sequencing to explore the genetic basis of the long hair trait at the genomic DNA level and found that several genes underwent strong selective sweeps. Among them, *Fgf5* has been reported to be closely related to hair growth in many species [8, 28–34]. It encodes the FGF5 protein, which functions at late anagen to control catagen entry on time [9]. Increasing evidence shows that genetic variants in *Fgf5* underlie hair length regulation, such as deletion, duplication and missense mutations [8, 28–37]. A series of allelic mutations in *Fgf5* resulted in Angora mice showing excessively longer fur formation due to a prolonged anagen phase [8]. In humans, a missense mutation in *Fgf5* converts a conserved tyrosine at residue 174 to histidine (p. Y174H), which is predicted to impact the function of FGF5, consequently causing abnormal hair growth [29]. Five missense mutations in *Fgf5* have also been identified to be associated with hair length variation in cats [28, 35, 36]. A 1-bp deletion in *Fgf5* is responsible for the male-dominant long hair phenotype in Syrian hamster. The deletion leads to a truncated abnormal FGF5, which enhances prolonged anagen and/or retards catagen induction [30]. In addition, previous studies on long-haired donkey, dog and human have raised a prediction that receptor binding capacity of FGF5 might be impacted by genetic mutations in *Fgf5* [29, 31, 32, 37]. In donkey, a frameshift deletion and a nonsense mutation exist in *Fgf5*, which led to stop codons at positions 159 and 82, respectively. The two truncated FGF5 proteins were predicted to lack the critical β strands that are necessary for the interaction between FGF5 and its receptor [31]. In dogs, a series of allelic mutations in *Fgf5* were associated with the long-hair phenotype [32, 37]. Especially, the p.C95F mutation is involved in hydrogen bonding and is directly adjacent to a conserved Tyr whose hydrogen bonding is probably critical to protein/receptor binding specificity [37]. Claire et al. also predicted that the p. Y174H mutation in humans is likely to impact the function of FGF5 such that it would either fail to bind to and activate its receptor, FGFR1, or do so at significantly reduced levels [29]. In this study, we discovered that a homozygous mutation (T19234C) was conserved in *Fgf5* exon 3 of Angora rabbits, which led to a missense mutation at residue 191 (p. L191S). The structural prediction of FGF5 showed that L191 was adjacent to the conserved ligand binding sites V94, N192, K193, K198, H209, V210 and S211. Moreover, L191 interacted with one of the ligand binding residues, S211. Therefore, we speculated that substitution of the hydrophobic leucine with a hydrophilic serine at residue 191 might impact the receptor binding ability of FGF5. Moreover, this speculation was further confirmed by a co-IP assay using FGF5 and mu-FGF5 plasmids. This demonstrates that the T19234C mutation is critical for Angora rabbits to develop their long hair trait and that the mutation weakens the interaction between FGF5 and FGFR1, a mandatory step to inhibit hair growth.

In addition to the *Fgf5*, other candidate genes identified in this study (*Dusp1*, *Ihh*, *Fam134a*, *Map3k1*, and *Spata16*) also may contribute to the long hair trait in Angora rabbits. It has been reported that DUSP1 and MAP3K1 proteins are involved in high-quality brush hair in the Yangtze River Delta White Goat [38]. DUSP1 regulates the growth and development of hair follicles. MiR-101 promotes the proliferation of hair follicle stem cells by targeting DUSP1[39]. Knockdown MAP3K1 expression inhibited the proliferation of hair follicle stem cells, which might regulate goat superior-quality brush hair traits [40]. MiR-31-5p significantly improved hair follicle stem cell proliferation and inhibited cell apoptosis by upregulating MAP3K1 level [41]. *Dusp1*, *Map3k1*, *Spata16*, and *Fgf5* are included in MAPK signalling pathway. *Ihh* and *Fam134a* participate in the Hedgehog signalling pathway. Both MAPK and Hedgehog signalling pathway are known to be involved in the regulation of hair growth cycle by promoting the proliferation and differentiation of hair-follicle stem cells, as well as increasing the production of growth factors [16–22]. In this study, 6, 2, 5, 1, 8 exonic SNP loci were identified in *Dusp1*, *Ihh*, *Fam134a*, *Map3k1*, and *Spata16*, respectively (Table S2), which may be related to the growth regulation of rabbit hair. We will further explore their functions on regulating rabbit hair length in the future study.

## Conclusions

In conclusion, by performing selective sweep analysis based on whole-genome sequences, we identified several candidate genes associated with the long hair trait in Angora rabbits. Among them, *Fgf5* is a well-established hair growth regulation gene. We discovered that a nonsynonymous mutation within *Fgf5* was conserved in Angora rabbits and that the mutation impaired the binding ability of FGF5 to its receptor FGFR1. In future study, further research will be carried out to investigate SNP in the additional candidate genes relative to their contribution to the long hair phenotype. Additionally, larger samples sizes that reflect the diversity of the breeds is needed to further quantify the LD patterns in the breeds discussed herein. These studies will provide valuable resources for future research on Angora rabbit breeding and insight into the mechanisms of artificial selection.

## Methods

### Sample collection

For whole genome sequencing, a total of 18 EDTA-blood samples of female rabbits, including 6 Angora rabbits, 6 New Zealand rabbits and 6 Rex rabbits, were collected from farms located in Shaanxi province of China. The samples were sent to Novogene Bioinformatics Institute (Beijing, China) for genome sequencing. To validate the specific SNP locus by Sanger sequencing, EDTA blood samples of an additional 135 rabbits (Angora *n* = 52, New Zealand *n* = 43 and Rex *n* = 40) were collected from Shaanxi, Shandong, Jiangsu and Anhui provinces of China. Following sampling, the rabbits were returned to their owners. There is no direct and collateral blood relationship within the last three generations among the sampled individuals. All research involving animals was approved by The Laboratory Animal Administration Committee of Xi’an Jiaotong University (Permit No. 2015–1317) and carried out according to the Guidelines for Animal Experimentation of Xi’an Jiaotong University and the Guide for the Care and Use of Laboratory Animals published by the US National Institutes of Health (NIH Publication No. 85–23, revised 2011).

### Library construction and sequencing

Genomic DNA was extracted from the EDTA-blood samples using the standard phenol‒chloroform protocol. Before library construction, agarose gel electrophoresis was performed to ensure the completeness and purity of the DNA, and OD260/280 was detected at 1.8 ~ 2.0. A total amount of 1.5 μg genomic DNA was used as input material for the DNA sample preparations. Qualified DNA was randomly cut into 350-bp fragments using a hydrodynamic shearing system (Covaris, Massachusetts, USA). Ended repair, added A-tail, ligation of paired-end adaptors, purification and PCR amplification with 350-bp inserts were sequentially processed to construct a paired-end sequencing library according to the manufacturer’s specifications (Illumina). Sequencing was performed on the Illumina HiSeq2500 platform, and 125 bp paired-end reads were generated with an insert size of approximately 350 bp. Raw FASTQ sequences have been deposited in the NCBI Short Read Archive under BioProject accession number PRJNA862254.

### Sequence quality checking and filtering

First, to ensure that reads were reliable and without artificial bias (low-quality paired reads, which mainly resulted from base-calling duplicates and adapter contamination) in the following analyses, raw data (raw reads) in fastq format were first processed through a series of quality control (QC) procedure in-house C scripts. QC standards are as follows: (a) Removing reads with ≥ 10% unidentified nucleotides (N); (b) Removing reads with > 50% bases having phred quality < 5; (c) Removing reads with > 10 nt aligned to the adapter, allowing ≤ 10% mismatches; (d) Removing putative PCR duplicates generated by PCR amplification in the library construction process (read 1 and read 2 of two paired-end reads that were completely identical). Second, high-quality paired-end reads were mapped to the rabbit reference genome sequence (ftp://ftp.ensembl.org/pub/release-78/fasta/oryctolagus_cuniculus/dna/) using BWA (Burrows‒Wheeler Aligner) software v0.7.8 [42, 43] with the command ‘mem -t 4 -k 32 –M -R’. SAMtools software v1.19 [44] (settings: –bS –t) was used to convert and index the mapping results to BAM files. In addition, potential PCR duplications were removed using the SAMtools command “rmdup”. If multiple read pairs had identical external coordinates, only the pair with the highest mapping quality was retained. The sequencing depth, genome coverage and other information of each sample are recorded in Table 1.

### Single nucleotide polymorphism (SNP) calling

After alignment, we performed SNP calling on a population scale for the three groups (6 Angora, 6 New Zealand and 6 Rex rabbits) using a Bayesian approach as implemented in the package SAMtools [45]. The genotype likelihoods from reads for each individual at each genomic location were calculated, and the allele frequencies were also estimated. The ‘mpileup’ command was used to identify SNPs with the parameters ‘-m 2 -F 0.002 -d 1000’. Then, to exclude SNP calling errors caused by incorrect mapping or InDels, only high-quality SNPs (coverage depth ≥ 4 and ≤ 1,000, RMS mapping quality ≥ 20, distance between adjacent SNPs ≥ 5 bp, no InDel present within a 3 bp window and missing ratio of samples within each group ≤ 50%) were kept for subsequent analysis.

### Linkage disequilibrium (LD) and phylogeny analyses

To estimate and compare the pattern of LD for different breeds, the squared correlation coefficient (r^2^) between pairwise SNPs was computed using Haploview v4.269 software [46]. Parameters in the program were set as ‘-n -dprime -minMAF 0.01’. The mean r^2^ value was calculated for pairwise markers in a 500-kb window and averaged across the whole genome. We found differences in the rate of decay and level of LD value, which reflected variations in population demographic history and effective population size (Ne) among breeds/populations. We also conducted principal component analysis (PCA) to evaluate genetic structure using GCTA software v1.24.2 [47].

### Selective sweep analysis and functional enrichment analysis

To identify genome-wide selective sweeps associated with the long hair trait, a sliding window approach (100 kb windows with 10 kb increments) was applied to quantify the polymorphism levels (heterozygosity, Hp, pairwise nucleotide variation as a measure of variability) and genetic differentiation (Fst) between Angora rabbit and the short-haired rabbit breeds (Rex and New Zealand). At each detected SNP position, we counted the numbers of alleles corresponding to the most and least frequently observed alleles (nMAJ and nMIN, respectively) in each pool. Hp for each window was calculated by the equation Hp = ∑nMAJ*∑nMIN /(∑nMAJ + ∑nMIN)^2^ [48]. Individual Hp values were then Z-transformed as follows: ZHp = (Hp-μHp)/σHp. The genome-wide distribution of the Fst value was calculated by VCFtools v0.1.14 [42] and Z-transformed, ZFst = (Fst-μFst)/σFst. We considered the windows with the top 5% ZFst and ZHp simultaneously as candidate outliers under strong selective sweeps. All outlier windows were assigned to corresponding SNPs and genes. To better understand the enriched pathway of the identified candidate genes, the Kyoto Encyclopedia of Genes and Genomes (KEGG) pathway was performed using KOBAS v2.0 [49, 50]. *P* values were calculated by hypergeometric test. The pathways were considered significantly enriched by genes when their Benjamini–Hochberg adjusted *P* values < 0.05.

### Sequence Validation

To validate the nonsynonymous nucleotide substitution in exon 3 of *Fgf5*, the genomic DNA of an additional 135 rabbits was extracted using a TIANamp Genomic DNA kit (TIANGEN Biotech), followed by amplification of the *Fgf5* partial fragment (containing the specific site) using 2 × Accurate Taq Master Mix (Accurate Biotechnology) and the following *Fgf5* primers: Fgf5-F1 (5’- CCTATGCCTCAGCAATACATAGAACT -3’) and Fgf5-R1 (5’- ATCCAAAGCGAAACTTGAGTCTG -3’). Amplified PCR products were purified and then sequenced by Sangon Biotech (Shanghai, China). The BioEdit program (version 7.0.9.0) [51] was used to perform sequence alignment analysis.

### Functional predictions of nonsynonymous mutations

The effect of the missense mutation (p. L191S) on FGF5 protein function was predicted using two functional mutation prediction programs: PROVEAN (http://provean.jcvi.org/seq_submit.php) [11] and PolyPhen-2 (http://genetics.bwh.harvard.edu/pph2/) [12], with the default cut-off values. The rabbit FGF5 protein sequence (UniProtKB-G1T394) was used as a query in the PROVEAN program. Because PolyPhen-2 specializes in analysing human SNPs, we made a corresponding amino acid substitution in the human FGF5 sequence (UniProtKB-P12034) based on the result of the sequence alignment between humans and rabbits and then ran the web application of PolyPhen-2 using the sequence. The prediction ‘Deleterious’ by PROVEAN was regarded as a function-altering mutation. The prediction ‘probably damaging’ with an output score close to 1 by PolyPhen-2 was also regarded as a damaging mutation.

In addition, the three-dimensional structure of the FGF5 protein was predicted by I-TASSER v4.2 [13]. Homology modelling was based on its homologue, FGF1 (PDB ID: 3OJV) [52]. Using Bio3D [53], an interactive tool for comparative analyses of protein structures, the best model was selected based on structural similarities, and a fitting procedure was conducted. Ligand binding sites and active sites of FGF5 were predicted by I-TASSER. PyMOL v2.5 [54] was used for visualization.

### Co-immunoprecipitation (Co-IP)

The plasmids of FGF5 (XM_008267686) and FGFR1 (XM_008273980) were purchased from GenScript (Nanjing, China). The mutant plasmid of FGF5 (mu-FGF5) was also constructed by GenScript. Nucleotide 572 within the coding sequence of *Fgf5* (c.572) corresponds to the 19,234 site within the whole gene sequence of *Fgf5*. Therefore, in the mu-FGF5 plasmid, c.572 was substituted with cytosine, which led to the leucine at amino acid 191 being mutated to a serine. Both FGF5 and mu-FGF5 are equipped with a C-terminal DYKDDDDK tag (Flag tag), which can be recognized and bound by an anti-Flag antibody. HEK293T cells were co-transfected with FGFR1 and FGF5 or mu-FGF5 plasmids. Forty-eight hours later, the cells were lysed with lysis buffer (50 mM Tris, pH 7.4, 150 mM NaCl, 0.2 mM EDTA, 2 mM EGTA, 0.5% Igepal CA-630, 10% glycerol, 1 mM sodium vanadate) supplemented with protease inhibitor (4693132001, Roche, Basel, Switzerland). The lysate was clarified by centrifugation at 14,000 X g for 5 min at 4 °C. The supernatant was transferred to a new tube and incubated with Anti-DYKDDDDK (Flag) IP Resin (L00425, GenScript, Nanjing, China) overnight at 4 °C. The final pellet from IP was subjected to elusion with 2 × Laemmli sample buffer (1610737, Bio-Rad, Hercules, USA) for 5 min at 95 °C. The IP samples were subjected to immunoblotting with antibodies against FGFR1 (WL01170, Wanlei bio, Sheyang, China) and Flag (66008, Proteintech, Wuhan, China).

### Immunoblotting

Protein samples were separated by sodium dodecyl sulfate–polyacrylamide gel electrophoresis (SDS-PAGE) and analysed by immunoblotting as described previously [55]. The separated proteins were transferred onto PVDF membranes and probed with antibodies against FGFR1, Flag and α-tubulin (ab15246, Abcam, Cambridge, UK). The antibodies bound on the membrane were detected with secondary antibodies conjugated with horseradish peroxidase (31460, Thermo Fisher Scientific, Rockford, USA), followed by visualization with a chemiluminescence substrate. The luminescence signal was recorded digitally by using a Chemi-Doc XRS imaging system (Bio-Rad). Digital image acquisition and analysis were conducted using the Quantity One program (Bio-Rad).

## Supplementary Information

**Additional file 1.** The entire original pictures of blots in Figure 5.

**Additional file 2. Table S1.** KEGG enrichment analysis of the 174 identified candidate genes.

**Additional file 3:Table S2.** Exonic SNPs of *Dusp1*,* Ihh*, *Fam134a*, *Map3k1* and *Spata16.*

## Data Availability

The datasets generated and/or analysed during the current study are available in the NCBI Short Read Archive repository, accession number PRJNA862254. All data generated or analysed during this study are included in this published article and its supplementary information files.

## Abbreviations

SNP: Single-nucleotide polymorphism
Fgf5: Fibroblast growth factor 5
Dusp1: Dual specificity phosphatase 1
Ihh: Indian hedgehog homolog
Fam134a: Family with sequence similarity 134 member A
Map3k1: Mitogen-activated protein kinase kinase kinase 1
Spata16: Spermatogenesis associated 16
FGFR1: Fibroblast growth factor receptor 1
MAPK: Mitogen activated kinase-like protein
