# Supplementary figures and images for: A homozygous missense mutation in the fibroblast growth factor 5 gene is associated with the long-hair trait in Angora rabbits
Source: BMC Genomics. 2023 Jun 2;24:298. doi: 10.1186/s12864-023-09405-2 (PMC10236585; doi:10.1186/s12864-023-09405-2)

## The entire original pictures of blots in Figure 5

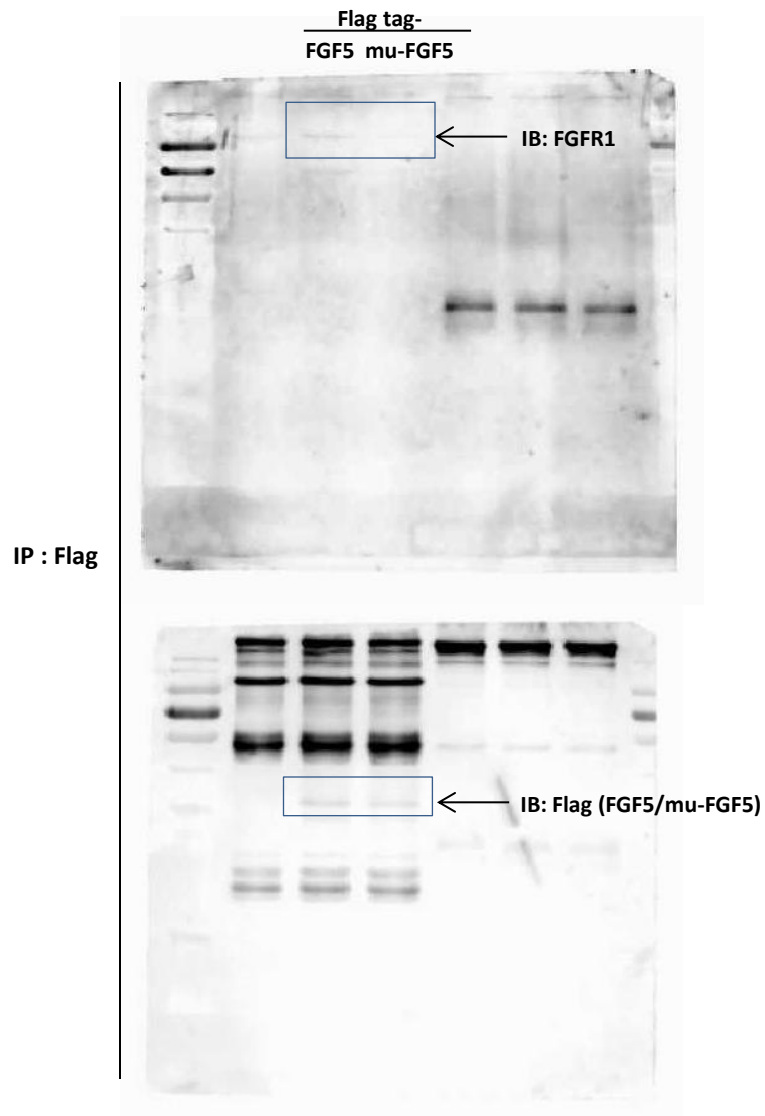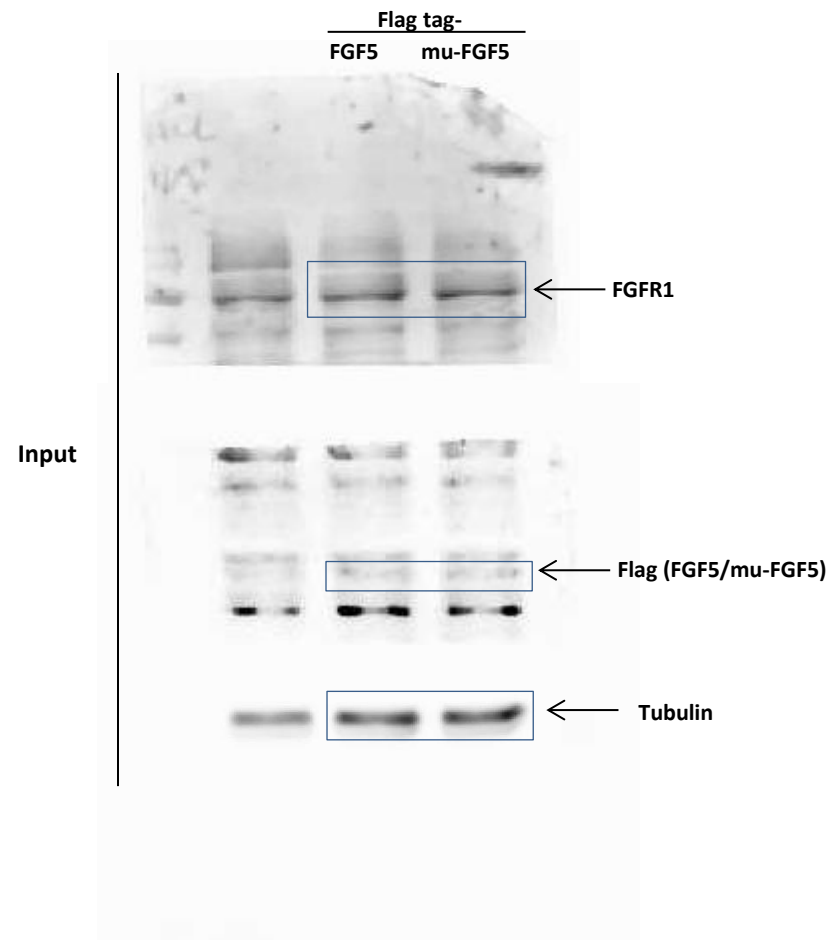

Supplement: Supplementary file 1 — Additional file 1. The entire original pictures of blots in Figure 5. [file 12864_2023_9405_MOESM1_ESM.pdf]
